# A rare presentation of delayed traumatic lumbar hernia after motor vehicle collision

**DOI:** 10.1093/jscr/rjac188

**Published:** 2022-05-31

**Authors:** Richard B Nguyen, Aakash A Trivedi, James Y Yang, Aakash A Anand, Jamshed Zuberi, Benjamin Rebein

**Affiliations:** General Surgery, St. George’s University School of Medicine, Paterson, NY, USA; General Surgery, St. Joseph’s University Medical Center, Paterson, NJ, USA; General Surgery, St. Joseph’s University Medical Center, Paterson, NJ, USA; General Surgery, St. George’s University School of Medicine, Paterson, NY, USA; General Surgery, St. Joseph’s University Medical Center, Paterson, NJ, USA; General Surgery, St. Joseph’s University Medical Center, Paterson, NJ, USA

**Keywords:** traumatic, lumbar, hernia, delayed

## Abstract

Traumatic abdominal wall hernia is defined as protrusion of bowel or an abdominal organ through a disruption of musculature and fascia following a severe blunt trauma. We report a case of a patient who had a delayed presentation of a traumatic, superiorly located paralumbar hernia months after the initial admission.

## INTRODUCTION

Traumatic abdominal wall hernia (TAWH) is defined as protrusion of bowel or an abdominal organ through a disruption of musculature and fascia following a severe blunt trauma. The most common causes of this rare phenomenon are motor vehicle collisions (MVC) followed by handlebar injuries, motorcycle accidents and falling [1]. TAWH make up 1% of trauma cases according to Burt *et al*. [2]. Seventy-three percentage of TAWH are present at admission, while the remainder are diagnosed afterward [1, 2]. TAWH typically present ventrally; however, rarely these hernias can present as lumbar hernia. According to Burt *et al*., there have been 66 reported cases of traumatic lumbar hernias reported in English literature since 1906 [2].

## CASE PRESENTATION

We present a case of a 27-year-old female with a history of an MVC that presented with abdominal pain and nausea for 2 days. Three months prior, she was the restrained driver when her car was hit on the driver’s side. X-ray imaging demonstrated a left hemothorax, left open fractures of the humerus and a left proximal femoral fracture. Computed tomography (CT) yielded a left apical pneumothorax, bilateral pulmonary contusions and a left-sided pelvic retroperitoneal hematoma. Along with surgical fixation for her fractures, she was admitted into the surgical intensive care unit (SICU) for monitoring. She was discharged 1 month later with no complications. She presented 2 months later with abdominal pain. CT scans suggested a partial small bowel obstruction (SBO). She was admitted, treated with bowel rest and was discharged 9 days later.

**Figure 1:**
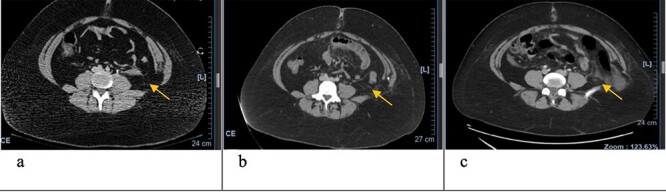
Axial CT scan with arrows pointing to internal and external oblique defects in June (**a**), August (**b**) and September (**c**); panel (a) shows rupture between internal and external oblique muscles at the attachment point of the quadratus lumborum; panel (b) shows fat stranding, suggesting inflammation of the epiploic appendage, and may be an early sign of delayed traumatic wall hernia; panel (c) shows herniation through the muscular defect.

**Figure 2:**
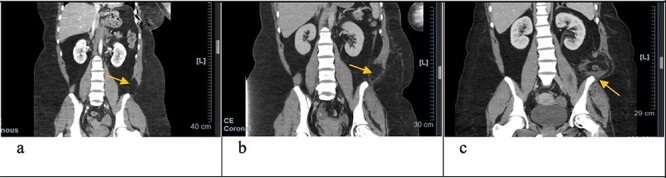
Coronal CT scan with arrows pointing to internal and external oblique defects in June (**a**), August (**b**) and September (**c**); panel (a) shows rupture between internal and external oblique muscles at the attachment point of the quadratus lumborum; panel (b) shows fat stranding, suggesting inflammation of the epiploic appendage, and may be an early sign of delayed traumatic wall hernia; panel (c) shows herniation through the muscular defect, with the descending colon without obstruction.

During her last admission, she experienced lower abdominal pain with nausea for 2 days. The abdomen was soft, obese with mild tenderness and guarding on palpation without rigidity or a hernia. CT scans suggested ileitis, omental adhesions, SBO and an inferior lumbar hernia. She underwent laparoscopic surgery for the ileitis, omental adhesions and repair of two mesenteric defects, while the lumbar hernia was opted to be handled electively; however, she did not desire additional surgery.

## DISCUSSION

TAWH arise from increased abdominal pressure, shearing forces and/or blunt forces on the abdomen, which cause a disruption of abdominal wall musculature and fascia. Blunt force results in the disruption of the abdominal wall muscles and fascia, allowing subcutaneous herniation of abdominal viscera through the defect, while the elasticity of the skin allows it to remain intact [3].

Lumbar hernias are rare, with 300 reported cases in English literature, 66 of which were traumatic lumbar hernias [2]. They can be congenital, acquired or through blunt trauma. Traumatic hernias are commonly due to increased intra-abdominal pressure leading to a muscle rupture or defect. They occur in two locations: Petit’s inferior lumbar triangle, and Grynfelt-Lesshaft’s superior lumbar triangle. In spontaneous lumbar hernias, the superior lumbar triangle is more commonly involved. In traumatic lumbar hernias, the inferior triangle is more common [3]. This could be due to the abrupt deceleration forces with increased intra-abdominal pressure caused by the seatbelt [4, 5], which rarely cause subcutaneous fat and muscular defects above the iliac crest [4], particularly in the case of obesity and high-riding seatbelts [6]. In our patient, the traumatic hernia was through the superior lumbar triangle. Complications of lumbar hernias may rarely include rarely SBO [7, 8].

Fat stranding is a common finding in late-onset TAWH. On CT, this appears as well-defined linear areas of increased attenuation caused by omental infarct, ischemia, perforation or infection [9]. In delayed TAWH, the grading of abdominal wall defects, inflamed stranding, hemorrhage or subtle defects at the time of admission may be potential indicators of future hernias [5].

In June, the patient’s CT imaging demonstrated a left posterolateral muscular defect of the fascia and external and internal obliques (Figs 1a and 2a). Follow-up CT in August showed an increase in size with noticeable atrophy of the muscles with fat stranding (Figs 1b and 2b). The atrophy may be due to the month-long period where she was immobile in the SICU. Muscle atrophy combined with the inciting event could be indicators for delayed TAWH, especially for those with prolonged hospital stays [3, 10]. When the hernia presented, CT imaging showed the appearance of fat and descending bowel (Figs 1c and 2c). We theorize that our patient’s MVC resulted in an initial defect or tear in the fascia, internal and external obliques. Her prolonged stay compounded the problem through muscle atrophy and peritoneal stretching. These factors, coupled with the activities of daily life, lead to the delayed presentation of traumatic lumbar hernia. Although lumbar hernias may rarely cause SBO, they may be indirectly linked.

## CONCLUSION

Traumatic lumbar hernias are due to high energy blunt traumas to the abdomen, particularly restrained drivers in MVCs due to shearing forces and/or increased intra-abdominal pressures. This is one of the first descriptions in literature showing a progression with imaging studies of how these hernias develop. In most cases, they are found at the time of admission. In our patient, we believe the delay in presentation may be due to an initial muscular and fascial defect of the abdomen, which was worsened by severe muscle atrophy from a prolonged hospital course. Additionally, our patient had a superior lumbar hernia, which is an uncommon presentation. CT evidence of fat stranding and muscular defect may have been an early indicator for delayed traumatic lumbar hernia.

## CONFLICT OF INTEREST STATEMENT

None declared.

## FUNDING

None.
